# Emergency Department Bedside Ultrasonography for Diagnosis of Acute Cholecystitis; a Diagnostic Accuracy Study

**Published:** 2018-01-20

**Authors:** Babak Shekarchi, Seyed Zia Hejripour Rafsanjani, Nima Shekar Riz Fomani, Mojtaba Chahardoli

**Affiliations:** 1 Department of Radiology, School of Medicine, AJA University of Medical Sciences, Tehran, Iran.; 2 Emergency Department, Besat Hospital, School of Medicine, AJA University of Medical Sciences, Tehran, Iran.; 3 Emergency Department, Firouzgar Hospital, Iran University of Medical Sciences, Tehran, Iran.

**Keywords:** Cholecystitis, acute, ultrasonography, diagnostic imaging, emergency service, hospital, emergency medicine

## Abstract

**Introduction:**

Using bedside ultrasound in diagnosing acute cholecystitis in the emergency department (ED) can save time, help the decision making process and allocate resources wisely. This study aimed to evaluate the diagnostic accuracy of bedside right upper quadrant (RUQ) ultrasonography in detection of acute cholecystitis.

**Method:**

In this diagnostic accuracy study, patients presenting to ED, suffering from RUQ pain in favor of acute cholecystitis underwent RUQ ultrasonography in emergency and radiology departments and interrater agreement between reports was calculated.

**Results:**

342 patients with the mean age of 53.92 ± 11.18 (20 – 83) years were studied (63.2% female). The number of patients with at least one sonographic finding of acute cholecystitis were 53 (15.50%) and 48 (14.00%) based on ED and radiology reports (Kappa = 0.826). Sensitivity, specificity, positive and negative predictive values, as well as positive and negative likelihood ratios of bedside sonography were 89.58 (95%CI: 76.55 – 96.10), 96.59 (95%CI: 93.63 – 98.29), 81.13 (95%CI: 67.58 – 90.11), 98.26 (95%CI: 95.77 – 99.36), 4.30 (95%CI: 2.42 – 7.62) and 0.017 (95%CI: 0.007 – 0.041), respectively.

**Conclusion:**

There was a very good agreement between ED and radiology departments’ sonography reports regarding the presence or absence of acute cholecystitis. Sensitivity and specificity of bedside RUQ sonography were 89.58 and 96.59, respectively.

## Introduction:

Around 10 to 20 percent of the normal population in the US have biliary stone but only 1 to 2 percent of them get symptomatic (1, 2). The most important complication of gallbladder stones is acute cholecystitis. The main signs and symptoms of acute cholecystitis are right upper quadrant (RUQ) pain, fever and Murphy sign (3). These findings guide the physicians towards proper diagnosis but they are not enough and they do not have adequate diagnostic yield to be precise (4, 5). Ultrasonography, hepatobiliary iminodiacetic with scintiography (HIDA) scan, and abdominal computed tomography scan are some of the available and helpful diagnostic tools with different accuracies in diagnosis of acute cholecystitis (6-8).

Ultrasound has been one of the most sensitive and specific modalities in acute cholecystitis diagnosis and has become the first line modality in many guidelines (8). It is vastly available, accurate and cost-beneficial and has been called “the 21^st^ century visual stethoscopes” (9-12). 

The most important sonographic findings of acute cholecystitis are gallbladder stone, increased wall thickness, and wall edema as well as fluid around the gallbladder (13, 14). 

Zenobi et al. showed the high positive predictive value of right upper quadrant (RUQ) ultrasonography in emergency settings for diagnosis of acute cholecystitis (15). Kendall et al. estimated the sensitivity 96% and specificity 88% of sonographic murphy sign for diagnosis of acute cholecystitis in an emergency setting (16). 

There was a recent massive improvement in point of care ultrasound science and technology, which made it useful for daily practice; however, there is still the question of accuracy. Therefore, the present study aimed to evaluate the diagnostic accuracy of bedside RUQ ultrasonography by trained emergency medicine residents or attending emergency physicians in detection of acute cholecystitis.

## Method:

***Study design and setting***

In this diagnostic accuracy study, patients presenting to emergency department of Firouzgar (a tertiary center for gastrointestinal diseases) and Besat Hospitals, Tehran, Iran, from 2015 to 2017, suffering from RUQ pain in favor of acute cholecystitis underwent bedside ultrasonography. Then, the accuracy of bedside ultrasonography performed by trained emergency residents or attending emergency physician was calculated, considering the radiology department reports as a standard. The study protocol was approved by ethics committee of AJA University of Medical Sciences and researchers adhered to all aspects of ethical practice and confidentiality of patients’ information. Informed consent was obtained before patients’ enrollment.

***Participants***

Patients with RUQ or epigastric pain suspected to acute cholecystitis who were brought to the mentioned emergency departments were enrolled using non-probability sampling techniques. Patients with history of biliary disease, jaundice, and cholecystectomy as well as intubated, pregnant and cases < 18 years old were excluded. 

***Investigation***

Following physical examination and history taking, each case was examined via bedside RUQ abdominal ultrasonography in the emergency department. All exams were either done by attending emergency physicians, expert in point of care ultrasound, or under their direct supervision by trained emergency residents. Ultrasonographies were done by a 2-5 MHZ curve transducer by an HM-70 Samsung device or an M-turbo Sonosite device. The examinations were started from the subcostal area. In case of no view from subcostal or intercostal window, the patient was asked to sit upright or rotate to the left lateral decubitus position. After examination, the sonographer filled out an online form consisting of 5 yes-no questions regarding the presence or absence of sonographic findings of acute cholecystitis as follows:

- Was there any stone in gallbladder?
- Was there gallbladder wall thickening > 3 millimeters?
- Was there any fluid around the gallbladder?
- Was there any wall edema in the gallbladder?
- Are the ultrasound findings in favor of acute cholecystitis?

Then, patients were transferred to the radiology department to be examined (usually by a radiology resident under observation of a radiology attending physician) and the answer of mentioned questions were extracted from radiology department digital imaging and communications in medicine (*DICOM*) system reports. 

Emergency residents participating in this study were among the 3^rd^ year emergency medicine residents who were trained in an hour-long theoretical class of ultrasound principles and knobology and 1-hour theoretical class of RUQ ultrasound including reviews of both normal and pathologic anatomy videos. Afterwards, they all had two hours of practice on a standard patient to have a hands-on practice. They did their first individual examination after 12 cases of direct supervision. Patients and their companions were blind to the sonography findings. 

***Data gathering***

Patients’ demographic variables (age, sex) as well as sonographic findings of emergency and radiology departments regarding presence or absence of acute cholecystitis sonographic findings were collected using a predesigned checklist by a trained medical doctor.

***Statistical analysis***

Data were analyzed using Statistical package for social sciences (SPSS) software version 20. All quantitative data were reported as mean ± standard deviation and qualitative data as frequency and percentage. For measuring inter-rater agreement between radiology and emergency departments’ reports Cohen's kappa coefficient was calculated. In this study kappa coefficient < 0.20 was considered as poor, 0.21 - 0.40 as fair, 0.41 - 0.60 as moderate, 0.61 - 0.80 as good, and 0.81 - 1.00 as very good strength of agreement.

Screening performance characteristics of emergency department’s RUQ sonography in diagnosis of acute cholecystitis were calculated using VassarStats medical software, considering radiology ward reports as standard test. 

## Results:

342 patients with the mean age of 53.92 ± 11.18 (20 – 83) years were studied (63.2% female). 179 (52.3%) ED sonographies were done by emergency physicians and 163 (47.7%) by trained 3^rd^ year emergency medicine residents. 

53 (15.50%) patients had at least one sonographic finding of acute cholecystitis based on ED reports, while radiology department reported the presence of these findings for 48 (14.00%) cases (Kappa = 0.826). Frequency of each sonographic finding in ED and radiology department reports is shown in table 1. The highest and lowest agreement between ED and radiology departments were regarding the presence of stone (Kappa: 0.884) and wall edema (Kappa: 0.343), respectively.

Sensitivity, specificity, positive and negative predictive values, as well as positive and negative likelihood ratios of ED sonography for screening of cholecystitis were 89.58 (95%CI: 76.55 – 96.10), 96.59 (95%CI: 93.63 – 98.29), 81.13 (95%CI: 67.58 – 90.11), 98.26 (95%CI: 95.77 – 99.36), 4.30 (95%CI: 2.42 – 7.62) and 0.017 (95%CI: 0.007 – 0.041), respectively. Table 2 summarizes the screening performance characteristics of ED bedside sonography for 4 sonographic findings of acute cholecystitis, separately.

**Table 1:** Frequency of each sonographic finding in emergency and radiology department reports

**Finding**	**Department n (%)**		**Kappa**
	**Emergency **	**Radiology**	
Gallstone	72 (21.1)	69 (20.2)	0.884
Wall thickness>3mm	44 (12.9)	41 (12.0)	0.745
Fluid around the gallbladder	22 (6.4)	22 (6.4)	0.660
Wall edema	12 (3.5)	10 (2.9)	0.343
Acute cholecystitis	53 (15.5)	48 (14.0)	0.826

**Table 2:** Screening performance characteristics of emergency department bedside sonography for 4 sonographic findings of acute cholecystitis

**Characteristics**	Gallstone	Wall edema	Fluid	Wall thickness
**True positive**	64	4	15	33
**False negative**	5	6	7	8
**False positive**	8	8	7	11
**True negative**	265	324	313	290
**Sensitivity**	92.7 (82.3-97.3)	40.0 (13.6-72.6)	68.2 (45.1-85.3)	75.0 (59.4-86.3)
**Specificity**	97.1 (94.1-98.6)	97.5 (95.1-98.9)	97.8 (95.3-99.0)	97.3 (94.6-98.7)
**PPV**	88.8 (78.7-94.7)	33.3 (11.3-64.6)	98.1 (45.1-85.3)	80.5 (64.6-90.6)
**NPV**	98.1 (95.5-99.3)	98.2 (95.9- 99.3)	97.8 (95.3-99.0)	96.3 (93.4-98.1)
**PLR**	8.0 (4.1-15.5)	0.5 (0.2-1.2)	2.1 (1.1-4.2)	4.1 (2.2-7.8)
**NLR**	0.01 (0.007-0.04)	0.01 (0.008-0.04)	0.02 (0.01-0.04)	0.04 (0.02-0.06)

PPV: positive predictive value; NPV: negative predictive value; PLR: positive likelihood ratio; NLR: negative likelihood ratio.

## Discussion:

Based on the findings of the present study, there was a very good agreement between ED and radiology departments’ sonography reports regarding the presence or absence of acute cholecystitis. Sensitivity and specificity of ED RUQ sonography were 89.58 and 96.59, respectively. 

Zenobi et al. in 2016 had their emphasis on murphy sign value in sonographic diagnosis of acute cholecystitis (15). Kendall et al. estimated the sensitivity of 96% and specificity of 88% , for sonographic murphy sign in diagnosis of acute cholecystitis in an emergency setting (16). Scruggs et al. conducted a study with a huge sample size of 1600 and calculated the sensitivity of 88% and the specificity of 84% for RUQ bedside ultrasonography (17). Some studies were focused on gallstones for diagnosis of acute cholecystitis (18, 19). Based on our findings, there was a very good agreement between emergency and radiology departments regarding detection of gallstones.

However, lower agreement in detecting some sonographic findings of cholecystitis such as gallbladder wall edema shows the necessity of continuous training in this regard. 

In point of care sonography the goal is finding an easy to do and easy to learn method without sacrificing the reliability. In 2009 Gaspari et al., in an attempt to specify a learning curve for bedside RUQ ultrasonography, concluded that after seven cases one will reach adequate image acquisition quality and technique and it will take around 25 cases to interpret the images reliably with a significant level of agreement with experts (20). In 2015 Blehar et al. analyzing a database of 52,468 scans in most important fields of point of care ultrasound concluded that reaching the plateau may take 90 cases (21). However, we should note that in their graphs the level of agreement, sensitivity and specificity was already high from the beginning. Our teaching method was really close to this study but combined with more hands-on sessions and a system of feedback after residents reported individually. Producing a guideline for reaching competency in RUQ ultrasound as a standard for evaluation and certification is recommended.

***Limitations***

The first limitation of this study was lack of a systematic image storing system; we had to export our data manually from our devices and it made the process more time-consuming. Lack of a gold standard as a scale for comparison was another problem. We don’t use HIDA scan in our centers, surgery is mostly done after passing the acute inflammatory phase, and computed tomography scan is used only in cases of suspected complications.

## Conclusion:

Based on the findings of the present study, there was a very good agreement between ED and radiology departments’ sonography reports regarding the presence or absence of acute cholecystitis. Sensitivity and specificity of bedside RUQ sonography were 89.58 and 96.59, respectively. Yet, lower agreement in detecting some sonographic findings of acute cholecystitis such as gallbladder wall edema shows the necessity of continuous training in this regard.
